# Esophageal cancer presenting as a brain metastasis: A case report

**DOI:** 10.3892/ol.2013.1436

**Published:** 2013-07-03

**Authors:** ALDO SPALLONE, CHIARA IZZO

**Affiliations:** 1 Section of Neurosurgery, Department of Clinical Neurosciences, Neurological Centre of Latium (NCL), Rome I-00178, Italy; 2 Department of Biomedicine, University of Rome ‘Tor Vergata’, Rome I-00173, Italy

**Keywords:** brain metastasis, esophageal cancer, unknown primary carcinoma

## Abstract

Carcinoma of unknown primary origin (CUP) is defined as the histological diagnosis of metastasis without the detection of a primary tumor. The incidence of CUP in all patients with a malignant disease has been reported to be between 3 and 15%. Esophageal cancer is the third most common type of cancer of the digestive tract and the seventh most common cause of cancer-related mortality worldwide. The overall incidence of the disease is highest in males >50 years old. Brain metastases have been reported in only 1.7–3.6% of all patients with different types of esophageal cancer. Brain metastasis as the presenting form of esophageal carcinoma is highly uncommon. The present study reports the case of a patient with an unknown primary tumor who presented with a cerebral metastasis, without extra-neurological symptoms. The CUP was subsequently diagnosed as an esophageal carcinoma.

## Introduction

The identification of the primary tumor in patients presenting with cerebral metastasis is mandatory in order to plan a proper treatment strategy. Unfortunately, in certain instances, the primary tumor remains occult even after a extensive and scrupulous diagnostic work-up (1,2).

Esophageal cancer was well described at the beginning of the 19th century, and the first successful resection was performed by Torek in 1913 (3). The overall incidence of the disease is highest in males >50 years old. Brain metastases have been reported in only 1.7–3.6% of all patients with different types of esophageal cancer (4,5), while brain metastasis as the presenting form of esophageal carcinoma is highly uncommon (6). The present study reports the case of a patient with a carcinoma of unknown primary origin (CUP) who presented with a cerebral metastasis, without extra-neurological symptoms. The CUP was subsequently diagnosed as a esophageal carcinoma. Written informed consent was obtained from the patient.

## Case report

A 69-year-old male patient was referred to the Neurological Centre of Latium (NCL) in July 2011 due to rapidly progressing right-hand apraxia and agraphia and recent reoccurring headaches. The patient was a heavy smoker (>40 pack years) and had a history of alcohol abuse. A physical examination indicated right, upper-limb prevailing hemiparesis [American Spinal Injury Association (ASIA) impairment scale, grade 1]. Furthermore, right dysmetria, adiadochokinesia and somatesthesic disturbance were present.

**Figure 1:**
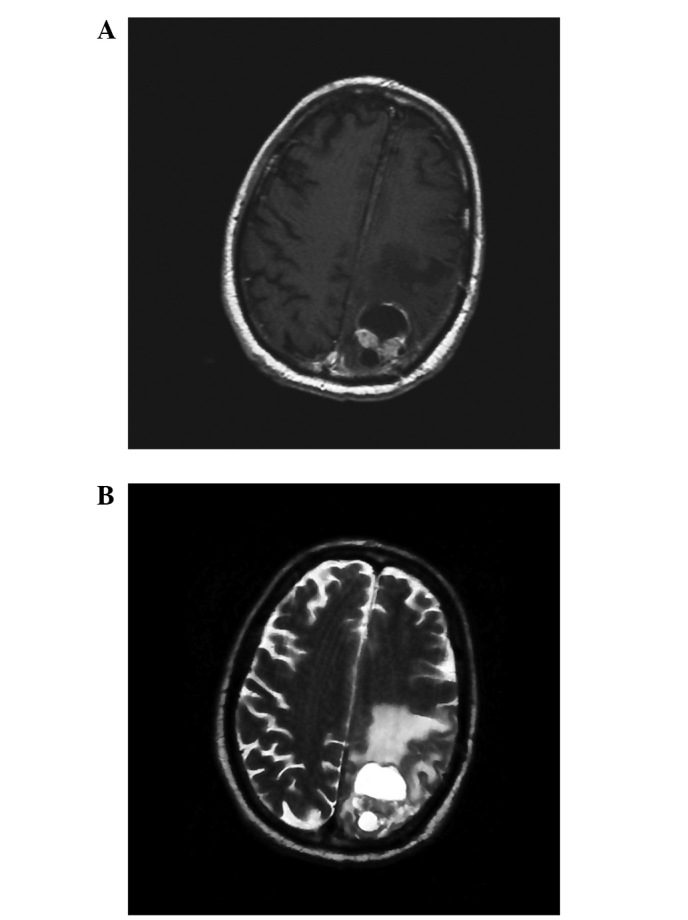
(A) T2 image of brain metastasis (axial view) showing a left parietal, oval-shaped, intra-axial mass lesion. High signal intensity indicates liquefactive necrosis and a small portion with signal intensity similar to grey matter representing a solid component. Perilesional edema is also observed. (B) Post-gadolinium T1 image of brain metastasis (axial view) indicating enhancement of the solid portion of the mass and linear enhancement of the wall.

An imaging work-up included computerized tomography (CT) scanning and brain magnetic resonance imaging (MRI), which indicated a low-density, contrast-enhancing subcortical lesion that was localized in the proximity of the left motor area, with perifocal edema and flattened sulci (Fig. 1). Whole-body completion CT scans were uneventful, thus leading to the diagnosis of a high-grade, primitive glial neoplasm.

**Figure 2:**
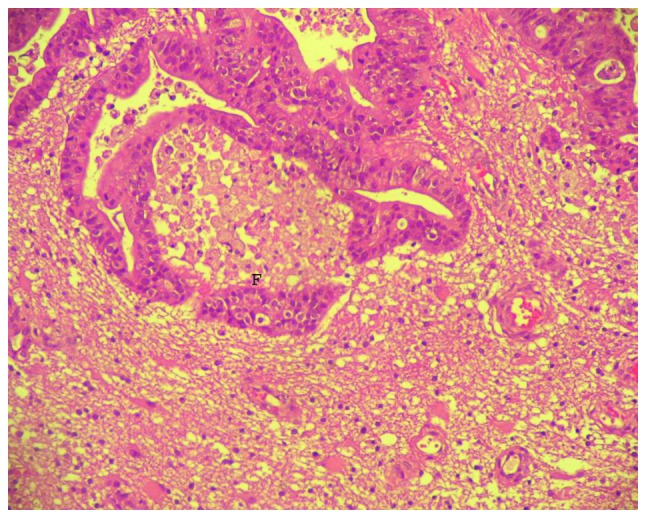
Histological assessment demonstrated a well-differentiated adenocarcinoma with anaplastic foci (F) and sublaminar areas of involvement. The staning pattern obtained following immunohistochemical analysis (cytokeratin monoclonal antibody staining; magnification, ×200) suggested a primary tumor arising from the digestive tract [thyroid transcription factor 1-negative (TTF1^−^) and carcinoembryonic antigen-positive (CEA^+^)].

The patient underwent microsurgical resection of the mass via a trans-sulcal approach. The histology was conclusive for a well-differentiated adenocarcinoma (Fig. 2). The staining pattern obtained following immunochemical analysis suggested a primary tumor arising from the digestive tract [thyroid transcription factor 1-negative (TTF1^−^) and carcinoembryonic antigen-positive (CEA^+^)].

Once discharged, the patient was scheduled for a complete oncological work-up that included a whole-body positron emission tomography (PET) scan and digestive tract endoscopy. The esophagogastroduodenoscopy revealed a flat, whitish area extending for ~2 cm in the longitudinal axis lying on the lower third of the thoracic esophagus, extending to the submucosa. No evidence of enlarged regional lymph nodes was present (cT2N0M1). The patient refused further surgical procedures and was scheduled for whole-brain and local radiotherapy and chemotherapy treatments. The protocol that was used consisted of a fractionated 50 Gy dose (2 Gy/day) and concurrent chemotherapy, including paclitaxel and cisplatin. An antiblastic therapy protocol was accomplished with a favorable course, despite an episode of non-fatal toxicity occurring subsequent to 2 months (transient leukopenia and pneumonia).

At the last follow-up at 14 months post-surgery, the patient was alive and well (Karnofsky index, >90). A neurological examination revealed amelioration of the motor function of the right hand and arm, with a positive impact on the quality of life physical domain, in spite of the development of a minor depressive syndrome. The patient was alive at 16 months after the initial diagnosis and, to date, no local or distant tumor recurrence has been documented.

## Discussion

The diagnosis of a brain metastasis is usually made during the routine follow-up examinations of patients with a known type of cancer. In the case of a CUP presenting with brain metastases, either a neurosurgeon or a neurologist are consulted prior to the oncologist. The incidence of brain metastasis of unknown primary origin is almost equal to that of cerebral metastases where the primary cancer is known (1,7). Moreover, a CUP will remain unknown for a period of time for the majority of the cases despite complete radiological and instrumental assessments (7).

Common contrast-enhancing malignant tumors of the brain are glioblastoma multiforme (GBMs), anaplastic astrocytomas (AAs), metastases and lymphomas, all of which are often characterized by similar conventional CT and MRI findings (8). However, metastatic tumors of the brain may exhibit different signal intensities on diffusion-weighted MRI (DWI) depending on their histology and cellularity. In fact, the signal intensity on DWI may predict the histology of the metastases (9), since well-differentiated adenocarcinomas tend to be hypointense, while small and large cell neuroendocrine tumors usually show hyperintensity (9,10). However, a study by Takeshima *et al*(11) suggested that the MRI findings of a cystic tumor with a thin enhancing rim may alert the clinician to the possibility of a metastatic brain tumor from the esophagus, particularly when a high-risk population is considered.

Patients with a newly detected brain mass and no history of other tumors, usually undergo extensive and expensive diagnostic testing to identify the primary neoplasm prior to the selection of a biopsy site. In this situation, a neurosurgical procedure may be considered as the most appropriate step to be taken in order to provide a definitive diagnosis, and also to avoid the unnecessary waste of time and resources. By contrast, it should be noted that esophageal cancer often lacks distinctive morphological characteristics leading to the potential unsuccessful identification of the site of origin by routine histological examination (12). Therefore, routine cancer screenings, such as whole-body PET and conventional diagnostic modalities (CT and/or MRI), are of fundamental value in detecting unknown primary tumors in inaccessible locations (13).

Esophageal cancer is a highly lethal disease with an extremely poor 5-year survival rate regardless of the stage of the disease. In 2008, esophageal cancer had an estimated annual incidence rate of 19.2/100,000 for males and 4.2/100,000 for females, who are exposed to a lower risk compared with males (14). At the time of diagnosis, ~50% of patients have metastatic disease and the majority of patients with localized esophageal cancer will develop metastases, despite potentially curative local therapy. The most common sites of distant recurrence, in order of frequency, are the lymph nodes (45%), liver (35%), lung (20%), cervical/supraclavicular lymph nodes (18%), bone (10%), adrenal (5%) and peritoneum (2%), while the incidence of brain metastasis is only 1.5% (15). Brain metastases have been reported in only 1.7–3.6% of all patients with different types of esophageal cancer (4,5,15). Patients with metastatic esophageal cancer have a median survival time of 6 months. The median patient age at the time of the diagnosis of brain metastasis was >60 years. The longest median survival time observed following the diagnosis of brain metastasis (9.6 months) occurred in patients with single brain lesions who underwent resection and received whole-brain radiotherapy (16,17).

There was a trend toward a worse survival in patients with liver metastases and patients in recursive partitioning analysis (RPA) class II–III vs. RPA class I. Moreover the reported 5-year survival rate ranges from 20 to 36% subsequent to intentionally curative surgery due to a high rate of either local or distant recurrence. The distant metastasis rate has been reported to be 26% within 20 months after radical surgery. Hematoxylin and eosin staining is one of the most effective predictors of survival in esophageal cancer due to the number of lymph node metastases detected using this technique (18,19).

The case reported in the present study is exceptional due to the patient’s prolonged, disease-free survival, the excellent response (to date) to non-surgical treatment of the primary disease and the clinical presentation resembling a brain glioma, which made the post-operative search for the primary tumor a challenge. Radical surgery on the brain lesion, even though located in an eloquent area, resulted in improvement to the presenting neurological deficits and represented the basis for a proper oncological assessment and successful management.

Esophageal carcinoma rarely presents with an isolated brain metastasis. In such cases, other than a careful assessment aimed to discover the unknown primary origin, removal of the lesion must be considered in order to treat the presenting symptoms and obtain a rapid histological diagnosis. Further studies are warranted in order to assess whether progression of the primary disease may be accelerated by post-surgical stress. This hypothesis should also be investigated against the potential benefits in terms of an improved quality of life and the advantages of a shorter diagnostic time.
